# Efficiency of Fabricated Adsorptive Polysulfone Mixed Matrix Membrane for Acetic Acid Separation

**DOI:** 10.3390/membranes13060565

**Published:** 2023-05-30

**Authors:** Kavita Pusphanathan, Hafiza Shukor, Noor Fazliani Shoparwe, Muaz Mohd Zaini Makhtar, Nor’ Izzah Zainuddin, Nora Jullok, Masoom Raza Siddiqui, Mahboob Alam, Mohd Rafatullah

**Affiliations:** 1 Bioprocess Technology Division, School of Industrial Technology, Universiti Sains Malaysia, Gelugor 11800, Malaysia; kavitapusphanathan@gmail.com (K.P.); muazzaini@usm.my (M.M.Z.M.); 2 Centre of Excellence for Biomass Utilization, Faculty of Chemical Engineering Technology, University Malaysia Perlis, Arau 02600, Malaysia; norajullok@unimap.edu.my; 3 Gold, Rare Earth and Material Technopreneurship Centre (GREAT), Faculty of Bioengineering and Technology, Universiti Malaysia Kelantan, Jeli Campus, Jeli 17600, Malaysia; fazliani.s@umk.edu.my; 4 Indah Water Konsortium, Lorong Perda Utama 13, Bukit Mertajam 14300, Malaysia; norizzahz@pop.iwk.com.my; 5 Chemistry Department, College of Science, King Saud University, Riyadh 11451, Saudi Arabia; mrsiddiqui@ksu.edu.sa; 6 Division of Chemistry and Biotechnology, Dongguk University, 123, Dongdaero, Gyeongju-si 780714, Republic of Korea; mahboobchem@gmail.com; 7 Environmental Technology Division, School of Industrial Technology, Universiti Sains Malaysia, Gelugor 11800, Malaysia

**Keywords:** mixed matrix membrane, acetic acid, polysulfone, ultrafiltration

## Abstract

The ultrafiltration mixed matrix membrane (UF MMMs) process represents an applicable approach for the removal of diluted acetic acid at low concentrations, owing to the low pressures applied. The addition of efficient additives represents an approach to further improve membrane porosity and, subsequently, enhance acetic acid removal. This work demonstrates the incorporation of titanium dioxide (TiO_2_) and polyethylene glycol (PEG) as additives into polysulfone (PSf) polymer via the non-solvent-induced phase-inversion (NIPS) method to improve the performance of PSf MMMs performance. Eight PSf MMMs samples designated as M0 to M7, each with independent formulations, were prepared and investigated for their respective density, porosity, and degree of AA retention. Morphology analysis through scanning electron microscopy elucidated sample M7 (PSf/TiO_2_/PEG 6000) to have the highest density and porosity among all samples with concomitant highest AA retention at approximately 92.2%. The application of the concentration polarization method further supported this finding by the higher concentration of AA solute present on the surface of the membrane compared to that of AA feed for sample M7. Overall, this study successfully demonstrates the significance of TiO_2_ and PEG as high MW additives in improving PSf MMM performance.

## 1. Introduction

Membrane technologies offer a novel opportunity in the bioprocessing industry for the removal of organic acids during upstream processing and for the recovery, purification, and concentration of products during downstream processing [1]. The lack of phase transition, reduced chemical requirements, small physical footprint, and low energy use in comparison to thermally typical methods are just a few of the reasons why they are so appealing [2]. This point of view is more relevant in the framework of water desalination, where the generation of water at the liquid energy nexus should require the least amount of energy [3]. In particular, the separation of acetic acid (AA) from water as well as other impurities using membrane technology satisfies the 12th goal of the Sustainable Development Goals (SDGs), which ensure sustainable consumption and production patterns by 2030. AA purification using membrane technology helps to achieve this goal as recovery is by means of pressure-driven membrane separation. Therefore, green AA separation and recovery will allow for its use in a wide range of applications, including the manufacture of paints, adhesives, and chemicals.

Recently, the “Fabrication of Adsorptive Mixed Matrix Membranes”, or “Green Technology”, has been applied to the separation and purification of liquids and gases. Nanoparticle-based membranes, also known as “nano-incorporation membranes”, are the building blocks of MMMs, which are also known as organic and inorganic nanocomposite materials. In contrast to traditional membranes, MMMs are constructed from an organic polymer that is embedded with organic fillers such as TiO_2_, PEG, zeolites, and MWCTs. Nanoparticles (NPs) can be blended into polymer membranes for a number of applications. In general, MMMs prepared by solution casting with filler materials result in dense composite membranes with improved mechanical strength and surface properties leading to enhanced performances, as reported by Lim et al. [3].

Interactions between the nanoparticular surface, polymer chains, and solvents are significant during membrane formation in order to achieve the desired membrane structure. These modifications to the membrane resulted in desired membrane structure of enlarged pore sizes and greater hydrophilicity [4]. Hydrophilic properties and the presence of nanoparticle functional groups also play a role in combating membrane fouling. Examples of polymers that can be used to create membranes include polyvinylidene fluoride (PVDF), polyamide (PA), polysulfone (PSf), and polyethersulfone (PES). PSf polymer, in particular, is frequently selected as a synthetic membrane for studying the influence of inorganic fillers on the resultant liquid separation properties. Inorganic fillers have previously proven to be successful in increasing the specific surface area, with tunable external surfaces and desirable physicochemical properties of MMMs that enhance its performance.

Optimal MMMs may depend on the choice of additives and the choice of method of application. To the former, titanium oxide (TiO_2_) and polyethylene glycol (PEG) represents two additives of choice that have been demonstrated to enhance the hydrophilicity and porosity of the MMM fabricated [5,6]. To the latter, dead-end ultrafiltration (UF) represents a pressure-driven approach for the separation of a low concentration of organic acid from water, comparable to reverse osmosis and nanofiltration. For purposes of AA separation, UF applicability has been demonstrated by Kresnowati et al. [7], specifically on the treatment of fermentation broth of oil palm empty fruit bunch (OPEFB), where the hydrolysate fermentation broth contains AA, xylitol, xylose, and other nutrient impurities. Prior to subsequent purification using electrodeionization (EDI), AA removal by UF at 1 bar transmembrane pressure resulted in 8.94 g/L left in the UF, corresponding to 35.96% from the original hydrolysate.

This study therefore explores the application of TiO_2_ and PEG in different formulations as additives to PSf for fabrication of MMM for use with UF under low pressure for recovery of AA. Membrane performance is evaluated by the resultant density and porosity under magnification, correlated to the subsequent degree of AA recovery.

## 2. Materials and Methods

### 2.1. Fabrication of Polysulfone Mixed Matrix Membrane (PSf MMMs)

PSf MMMs were fabricated using the non-solvent-induced phase-inversion method (NIPS). Prior to fabrication, the dope solution was prepared using the fixed formulations shown in Table 1. A fundamental step before using PSf polymer, it was dried in an oven for 20 h at 80 °C. In the oven, we avoided using combustible or dangerous vapor-producing materials. The PSf for each of the eight samples was then weighed and separated into separate containers. To make a proper dope solution, the additives should be mixed first in the solvent, then followed by the polymer. Following the formulations, all of the additives and solvent were weighed.

**Figure 1:**
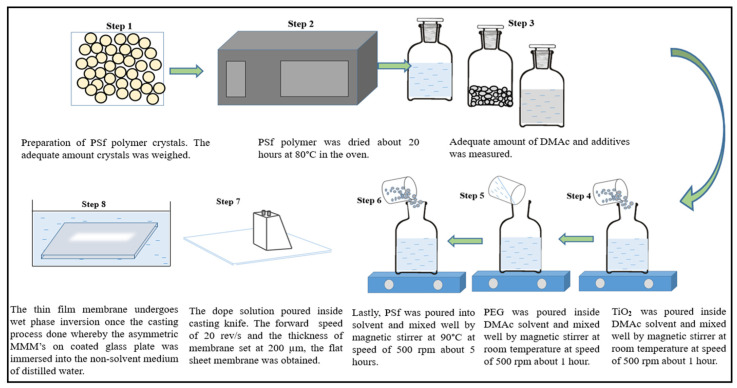
Simplified diagrammatic representation for the preparation of PSf MMMs [11].

Then, the dope solution was prepared by mixing the additive, which was TiO_2,_ in the solvent N-N-dimethylacetamide (DMAc) at room temperature using a magnetic stirrer. A hot plate was utilized to enhance the efficiency of nanoparticle dispersion in the solvent as shown in Figure 1. After that, PEG was added and dissolved into the solution and stirred continuously around 25 °C. Then, PSf was mixed in small amounts continuously at 500 rpm into the dope solution at 90 °C [8]. A thermometer was utilized to measure the mixing process at 90 °C. The casting solution needed to be mixed well under continuous agitation on the hot plate at 90 °C for 5 h [9]. The important rule of thumb during the making of dope solution was to avoid the solution being in contact with distilled water.

A casting knife was used during the fabrication process to spread 200 µm of dope solution over a glass plate. To cast the porous, thin-film membrane, the forward speed was set to 20 rev/s. After the casting process was completed, the thin-film membrane went through a 30 s dry–wet phase inversion. To facilitate solvent-free non-solvent exchange, the asymmetric flat-sheet PSf MMMs on a coated glass plate were submerged in a bath of distilled water. The resulting film membrane was dried at room temperature before being cut into the desired shape [10]. Finally, the membrane was dried at room temperature for 24 h.

### 2.2. Characterization of Fabricated Ultrafiltration Polysulfone Mixed Matrix Membranes (PSf MMMs)

Several membrane characterization methods were used to illustrate the characterization of ultrafiltration PSf MMMs in this section. Multiple options exist for defining the membrane’s structural morphology and functional properties. The characterization of membranes is seen as essential for both fundamental studies of membranes and the creation of useful membrane processes. Traditional membrane characterization techniques also included measuring pore size and pore size distribution. The characterization of membranes can be divided into two categories: physical properties and chemical properties.

#### 2.2.1. Physical Characterization

Scanning electron microscopy (SEM), contact angle (CA) evaluation, and porosity were used to assess the membrane’s physical properties, with a focus on morphology and pore size.

##### Scanning Electron Microscopy (SEM)

SEM was used to characterize the surface and volume structures of the membrane. It produces images of membrane samples by scanning their surfaces with a focused electron beam. SEM is used to examine membrane morphology and explain fouling mechanisms in terms of porosity and pore size distribution in terms of pore blockage. The plain view and cross-sectional view of the membrane were observed in this SEM analysis. The active part of the membrane was taken for analysis, and the membrane was subjected to a dead-end filtration test.

Firstly, the active part of membrane was cut into smaller pieces with 1 cm × 1 cm dimension for all the membrane samples, respectively. Similar measurements were fixed for both the plane view and cross-sectional view. After that, for the cross-sectional view analysis, the membrane sample was immersed in liquid nitrogen for 1 min and then fractured. The purpose of this freeze-drying method is to view the cross-section of a sample by imaging the sample’s interior cross-section of the membrane in SEM. As an initial step for the freeze-drying method, liquid nitrogen was poured into a thermos flask. Then, the membrane was stuck on the cardboard using cellophane tape so that the membrane was in a static position. From the thermos flask, an adequate amount of nitrogen liquid was poured into the beaker. Promptly, with the aid of forceps, the membrane was dipped in the nitrogen liquid. After a few seconds, the membrane can crack with a cracking sound. The cracked section will be sent for SEM analysis. When handling the nitrogen liquid, wear safety gloves and a face shield in order to avoid the splashing of it on body parts.

The double-sided carbon adhesion foil was used as a holder for the sample during the SEM analysis [12]. The membrane’s surface and cross-sectional area were then coated with a thin layer of platinum using sputter-coating under vacuum to neglect electrostatic charging, and an SEM test was performed. As a final step, the system generated images of membrane morphology.

##### Contact Angle (CA) Evaluation

The hydrophilicity of the membrane surface was determined using a contact angle measurement at room temperature [12]. First, the necessary equipment was assembled, including a syringe with a needle, a glass slide, cellophane tape, a protractor, colored ink, and a camera. The flat-sheet membrane sample was mounted on a glass slide with two slides to ensure that the upper surface of the membrane was looking upward as the first step in the contact angle evaluation. Following that, colored ink was dropped into the distilled water, and a syringe was used to suck the distilled water. After 5 s, the contact angle was measured by mounting 0.2 µL of distilled water onto the edge of the dry membrane’s surface. At a long working distance, a camera was used to capture the mounted droplet on the membrane, and the contact angle between the droplet and the substrate was measured. For each membrane sample, the contact angle evaluation was conducted 5 times, and the average of those readings was recorded. The measuring error that might be taken into consideration is the position of the camera, which should be well-centered accurately when capturing the mounted droplet on the membrane for further angle measurement. The same procedures were followed for each of the membrane samples. The contact angle value was determined by analyzing the result in the angle pro meter application. To avoid experimental error, each sample received an average of 10 readings, and the mean values were calculated.

##### Porosity

The porosity of a membrane can be determined using its dry weight. Distilled water was used to immerse the membrane [13]. The weight of the wet membrane was then measured after the excess wet membrane was removed with filter paper. The wet membrane was dried in a 25 °C oven for 10 h. Equation (1) was used to calculate the measured weight of the dry membrane.

(1) $$\varepsilon \left(\%\right)\frac{W_{W}-W_{d}}{W_{w}-\frac{W_{d}}{D_{w}}+\frac{W_{d}}{D_{p}}}\times 100\%$$

where ε is the membrane porosity, 𝑊*_w_* is the wet membrane weight (g), 𝑊*_d_* is the dry membrane weight (g), D_w_ is the pure water density (1.0 g/cm^3^), and D_p_ is the polymer density (1.37 g/cm^3^).

##### Mean Pore Size of Membrane

The average pore radius size was calculated using pure water flux (PWF) and porosity data (r_m_). The Guereout–Elford–Ferry equation [14] is used to calculate porosity data. The average pore radius size (r_m_) was determined using Equation (2).

(2) $$r_{m}=\frac{\sqrt{\left(2.9-1.75Porosity\right)8\eta l}Q}{Porosity\times a\times ∆P}$$

where η is the water viscosity (8.9 × 10^−4^ pa·s), *l* is the membrane thickness (m), Q is the pure water flux (m^3^/s), a is the area (m^2^), and ∆P is the operating pressure (3 bar).

#### 2.2.2. Chemical Characterization

The chemical characteristics of the membrane were evaluated via Fourier transform infrared spectroscopy (FTIR) and X-ray diffractometer (XRD), which mainly focus on the structure, chemical composition, and crystallinity of the membrane.

##### Fourier Transform Infrared Spectroscopy (FTIR)

The FTIR technique was used to determine the functional group on the membrane’s surface. FTIR spectroscopy is used to discover about the chemical interactions between molecules and thus learn about the surface chemistry of synthetic membranes. IR spectra are collected in a broad range, from 4000 cm^−1^ to 425 cm^−1^, by the FTIR spectrometer all at once. This is a huge benefit over the dispersive spectrometer, which can only measure intensity over a small range of wavelengths simultaneously. Attenuated total reflectance (ATR) was a sampling method that involved the use of light to determine the sample’s structure and chemical makeup. When it comes to FTIR spectroscopy, ATR is a popular sampling technique. The FTIR was linked to a 450 incidence angle diamond crystal and outfitted with an OM-NI-sample attenuated total reflection (ATR) smart accessory. On average, 32 scans were used to record each spectrum at a resolution of 4 cm^−1^.

##### X-ray Diffractometer (XRD)

The presence of PSf, TiO_2_, and PEG in the membranes’ polymeric network has been validated by X-ray diffraction analysis. In general, XRD peaks for the crystalline regions of membranes were investigated, as well as the effect of nanoparticles on crystal or amorphous membranes. Initially, an X-ray source, such as a cathode tube, is used to generate X-rays of a single wavelength frequency, which are then concentrated and fired at the sample, where they are refracted by the atoms and detected by the detector. The refracted X-rays have the same energy as the incident wave, which causes elastic scattering and allows the diffraction pattern to be observed [15]. A component’s diffraction beam is compared to a general reference database that depicts the purity and crystal properties of the component [16]. Cu Kα radiation will be used to perform the analysis on a Bruker X-ray diffractometer. The scanning rate was set to 100 to 600 (2-angle), Cu Kα X-rays with a wavelength of (λ) = 1.5406, and data with a step of 0.1972°. The data analysis was carried out with the help of the accompanying software (EVA and Expert). Bragg’s law was used to calculate the values for interplanar spacing, as shown in Equation (3), and the Debye–Scherrer equations were used to calculate polymer and additive crystallite size, as shown in Equation (4).

Bragg’s Law:

(3) $$\lambda =2d_{hkl}sin\emptyset$$

where λ is the wavelength of the X-ray source (0.15406 nm), $d_{hkl}$ is the interplanar spacing, and sin∅ is the diffraction angle in degrees (°).

Debye–Scherrer Equation:

(4) $$D=\frac{K\lambda }{\beta cos\emptyset }$$

where D is the particle diameter in nm, λ is the wavelength of the X-ray source (0.15406 nm), K is constant equal to 0.9, β is the full-width half maximum (FWHM) of the X-ray diffraction peak, and ∅ is the diffraction angle in degrees (°).

##### Membrane Permeation Test for Acetic Acid Removal

**Figure 2:**
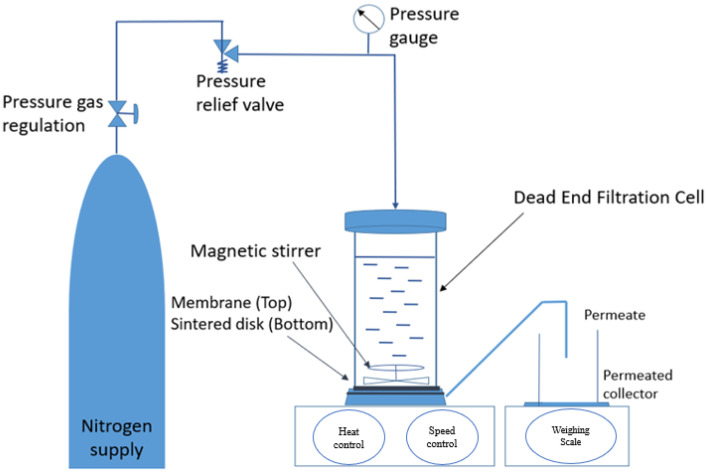
Schematic diagram of membrane permeation test using dead-end filtration unit.

The PSf MMMs’ performance was assessed using pure water flux (PWF), AA flux, and rejection. Figure 2 depicts the membrane permeation test setup. The dead-end filtration unit was linked to a nitrogen gas cylinder, which was used to test the membrane’s pure water and acetic acid flux under various pressures. At room temperature and pressures ranging from 3 bar, the membrane was tested. The dead-end filtration unit had a 5.1 cm cell diameter, a 22.4 cm height, and a 300 mL volume capacity with a Teflon-coated magnetic stir bar. The effective filtration area of the membrane was 14.6 cm^2^. In addition, 250 mL of diluted acetic acid was added to the stirred cell as a feed solution.

The membrane’s pure water flux was measured after about an hour. PWF was calculated using quantitative analysis, as shown in Equation (5).

(5) $$J_{WF}=\frac{V}{A_{m}t}$$

where J_WF_ is the pure water flux (L/m^2^·h), V is the permeate volume (L), A_m_ is the effective filtration area (m^2^), and T is the measurement time (h).

After pure water filtration, an hour of AA solution was brought to a pressure of 3 bar. The concentration of AA before and after filtration was determined using a UV-VIS spectrophotometer. Equation (6) was used to calculate the AA flux at twelve one-hour intervals.

(6) $$F_{AA}=\frac{V}{A_{m}t}$$

where F_AA_ is the AA flux (L/m^2^·h), V is the permeate volume (L), A_m_ is the effective filtration area (m^2^), and T is the measurement time (h).

For retentate flux, Equation (7) is as follows:

(7) $$R_{AA}=\frac{V}{A_{m}t}$$

where R_AA_ is the AA flux (L/m^2^·h), V is the retentate volume (L), A_m_ is the effective filtration area (m^2^), and T is the measurement time (h).

Moreover, the removal of AA as retentate was calculated using Equation (8).

(8) $$R_{AA}(\%)=\frac{{\left[AA\right]}_{in}-{\left[AA\right]}_{fi}}{{\left[AA\right]}_{in}}$$

where R_AA_ is the retentate of AA (%), [AA]_in_ (mg/mL) is the initial concentration of AA in the feed solution, and [AA]_fi_ (mg/mL) is the final concentration of AA.

#### 2.2.3. Fouling Resistance Evaluation

Fouling resistance was calculated using relative flux reduction (RFR), as shown in Equation (9).

(9) $$RFR\left(\%\right)=1-\frac{J_{AA}}{J_{WF}}$$

where RFR is the relative flux reduction, J_AA_ is the tested solution (AA solution) permeate flux (L/m^2^·h), and J_WF_ is the initial water flux.

The membrane was then cleansed with distilled water for 15 min before filtration resumed with the addition of pure water to the feed tank. For the second time, the PWF measurement was used to assess the flow recovery of the membrane. The second pure water flux lasted about an hour. The flux recovery of the membrane was calculated using Equation (10).

(10) $$FRR\left(\%\right)=\frac{J_{WF2}}{J_{WF}}\times 100\%$$

where J_WF2_ was the PWF after the washing step (L/m^2^·h).

#### 2.2.4. Concentration Polarization (CP)

The concentration of the solute that is retained on the surface of the membrane can be calculated by using Equation (11):

(11) $$C_{m}=\left(C_{b}-C_{p}\right)\exp\left(Pe\right)+C_{p}$$

where C_m_ is the accumulated solute near the membrane surface in (g/cm^3^), C_b_ is the solute concentration in the bulk feed solution in (g/cm^3^), C_p_ is the concentration of solute after permeation in (g/cm^3^), and exp Pe is the boundary layer Peclet number and is defined as Jδ/D. J is the volumetric flux through the membrane in (g/cm^2^·h), δ is the thickness of the mass transfer boundary layer in (cm), which is about 0.02 cm, and D is the diffusivity of the solute in (cm^2^/h).

Fick’s law was used to calculate the AA diffusivity in (cm^2^/h) as follows:

(12) $$J=-D\frac{dC}{dX}=-D\frac{C_{2}-C_{1}}{X_{2}-X_{1}}$$

where J is the retentate accumulated on membrane in (g/cm^2^·h), C_1_ is solute concentration in the bulk feed solution in (g/cm^3^), C_2_ is concentration of solute after permeation in (g/cm^3^), (X_2_ − X_1_) is the length of stirred cell, which is about 19.9 cm.

## 3. Results

Membrane separation technology approaches were applied via ultrafiltration UF PSf MMMs for the separation of acetic acid (AA). Asymmetric UF MMMs were developed successfully with new formulations of polymer, solvent, and additives. The characterization procedure was executed as the next step, followed by the ultrafiltration test. Evaluation of the antifouling properties was carried out as the final procedure. This segment covers all the output from the experimental procedures as follows:

i. To fabricate and characterize asymmetric polysulfone mixed matrix membranes (PSf MMMs);
ii. Determination of membrane performance and its efficiency based on flux and rejection of acetic acid (AA) solute;
iii. Interpretation of antifouling properties of fabricated MMMs as well as the correlation of concentration polarization (CP) on the surface of the membrane.

### 3.1. Fabrication and Characterization of PSf MMMs

#### 3.1.1. Formation of Fabricated Flat-Sheet PSf MMMs

The non-solvent-induced phase-inversion method was used successfully to fabricate asymmetric flat-sheet MMMs (NIPS). In the formulation, two types of additives were used: a constant 0.5 wt.% amount of TiO_2_ and different molecular weights of PEG. Using the casting knife, the thickness of the membrane was set constant for all samples at about 200 µm during fabrication. The MMMs were white in color, had a flat-sheet appearance, and were stored in filtered water for further perusal.

#### 3.1.2. Characterization of MMMs

For characterization procedures, the chemical and physical properties of the membrane were analyzed on the fabricated PSf MMMs in terms of structural, optical, and surface chemistry analyses through Fourier transform infrared spectroscopy (FTIR) to analyze the availability of functional group, contact angle measurement to measure the wettability of the membrane, followed by porosity, mean porosity, then morphological analysis performed using scanning electron microscopy (SEM), and average crystallite size of polymers and additives was observed via X-ray powder diffraction (XRD).

##### Fourier Transform Infrared Spectroscopy (FTIR)

**Figure 3:**
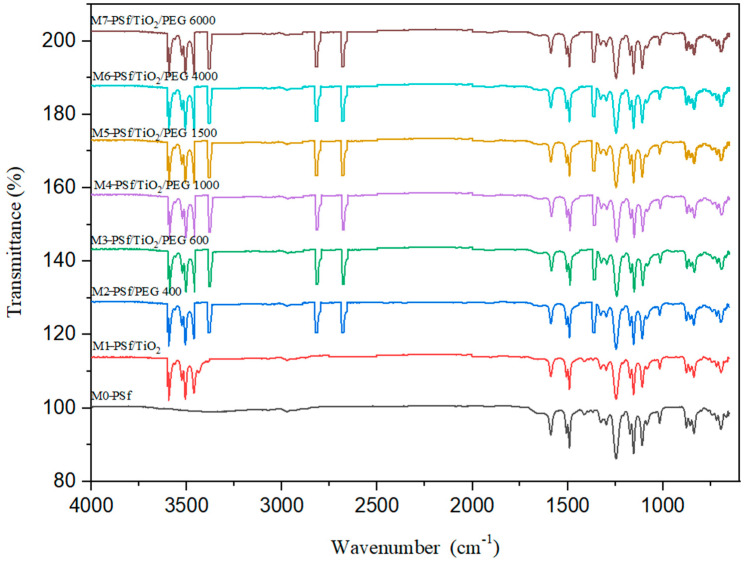
FTIR spectra of PSf MMMs from M0, M1, M2, M3, M4, M5, M6, and M7. Each IR Spectra line represented the functional groups found in membrane samples.

Chemical analysis of fabricated PSf MMMs was performed using Fourier transform infrared spectroscopy (FTIR). These were conducted between wavelengths ranging from 4000 cm^−1^ to 650 cm^−1^. Figure 3 depicts the FTIR spectra of pure PSf, PSf/TiO_2_, PSf/PEG 400, PSf/PEG 600/TiO_2_, PSf/PEG 1000/TiO_2_, PSf/PEG 1500/TiO_2_, PSf/PEG 4000/TiO_2,_ and PSf/PEG 6000/TiO_2,_ respectively.

FTIR is the most effective alternative approach for detecting membrane functional groups in general. The chemical structures of pure PSf, PSf/TiO_2_-blended PSf, and PSf-PEG-blended PSf were studied initially. The 1583 cm^−1^ and 1502 cm^−1^ bands are attributed to the strong reflection of benzene rings stretching for pure PSf, which was the M0. Sulfone C-SO_2_-C stretching is symmetric at 1149 cm^−1^, but asymmetric C-SO_2_-C stretching is detected at 1319 cm^−1^ and 1272 cm^−1^ [6]. The distinctive bands at 861 and 698 cm^−1^ are caused by aromatic C-H bending [17]. In general, the PSf layer’s absorption is defined by O=S=O values of 1000–1300 cm1 and C=C values of 1400–1600 cm^−1^ for the PSf membrane. Two additional minor bands at 1386 cm^−1^ and 1366 cm^−1^ are present for the PSf support, indicating the unique presence of methyl groups in PSf [18].

Furthermore, the PSf/TiO_2_ FTIR spectra show a narrow band centering around 500–600 cm^−1^ that is attributed to the TiO_2_ lattice’s bending vibration (Ti-O-Ti) bonds. The intermolecular interaction of the hydroxyl group in the PSf polymer chain with the TiO_2_ surface is responsible for the broad band at 3600–3400 cm^−1^. The functional groups are identical to those present in the PSf repeating units. The PSf/TiO_2_ membrane demonstrated the existence of oxygen-containing TiO_2_ groups, such as C-O spanning 1101 cm^−1^. Due to the presence of oxygen-containing groups in TiO_2_, spectroscopy reveals a peak at 600–1000 cm^−1^ for the blended PSf/TiO_2_ membrane, indicating successful Ti-O-Ti stretching. The high degree of agreement demonstrates that the PSf membrane created in the lab is genuine and that any contaminants or solvents that could interfere with how efficiently the membrane separates have been eliminated.

The FTIR spectra of the PSf/PEG blend membrane M2 reveal some band position fluctuations, showing that PSf and PEG interact. For pure PSF, the benzene ring stretching band is between 1583 cm^−1^ and 1502 cm^−1^, and for PSf/PEG, it is between 1585 and 1503 cm^−1^. Furthermore, the symmetric and asymmetric sulfone band locations changed to higher wavenumbers from 1149 cm^−1^, 1319 cm^−1^, and 1292 cm^−1^ to 1150 cm^−1^, 1320 cm^−1^, and 1294 cm^−1^, suggesting the presence of a PEG/PSf interaction [19]. When compared to pure PSf, the band position of C-H bending shifts to a higher wavelength range (from 861 cm^-1^ and 698 cm^−1^ for PSf and PEG, respectively, to 866 cm^−1^ and 704 cm^−1^), and its intensity decreases. A transesterification process between PSf and PEG alkoxide was proposed and investigated for the synthesis of polysulfone-poly (ethylene glycol) (PSf/PEG) amphiphilic block copolymers. When the PSf/PEG/TiO_2_ membrane was created, the PEG’s positively charged double C=C groups could interact with the TiO_2_’s OH group’s electron-drawing oxygen. This Lewis acid–base interaction enhances the polymer/filler interface, preventing non-selective voids [20]. Table 2 depicts the interaction that occurred in the PSf/TiO_2_/PEG MMMs. These interactions may influence the acetic acid permeation properties of the resultant membranes. The quality of the polymer membrane and the structure of the inorganic filler have a significant impact on acetic acid separation.

##### X-ray Diffractometer Analysis (XRD)

**Figure 4:**
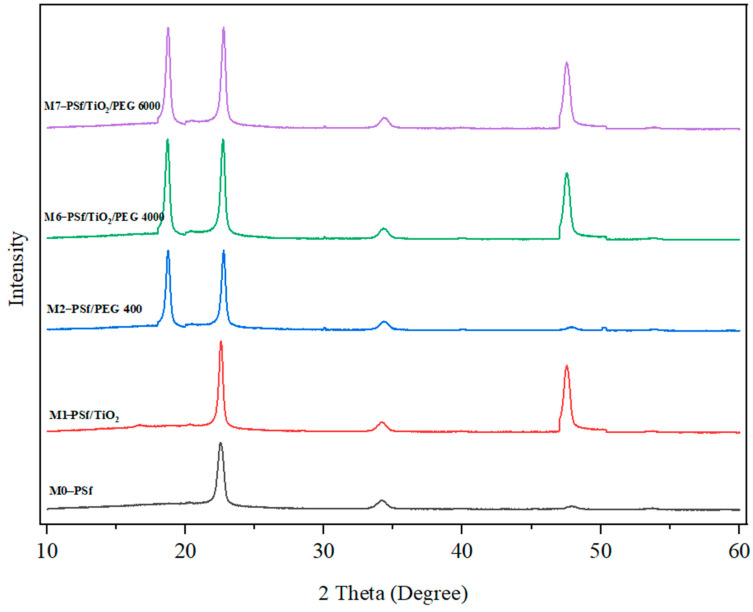
XRD patterns of M0, M1, M2, M6, and M7 membranes. Each XRD diffraction lines represented the membrane samples with independent formulations at different intensity.

The structure of the pure polysulfone, PSf/TiO_2_, PSf/PEG, and PSf/TiO_2_/PEG was studied by XRD diffraction. The primary fundamental of X-ray diffraction is to reflect toward the crystalline size of PSf, TiO_2,_ and PEG indicated by diffraction peaks. Four prominent diffraction peaks were observed in Figure 4.

The six prominent peaks observed at 2θ were 22.45°, 34.109°, 47.57°, and 19.10° as listed in Table 3. These peaks correspond to the face-centered cubic (FCC) of the polymer and PEG crystals as well as TiO_2_ powder [21]. Pure PSf polymer exhibited a broad peak at 22.45° and a narrow peak at 34.109°. The first peak in the spectrum was a wide peak caused by hydrogen bonding as well as dimethyl formation (DMAc) employed as a solvent [22]. This proof, which is based on the amorphous polymer’s XRD results, also exhibits a similar peak. This peak is caused by the introduction of groups along the chain that can form hydrogen bridge bonds between and within chains, which allows for the formation of a more regular structure and an emerging crystalline behavior in the PSf [23].

From M2, M6, and M7, the XRD of PEG is observed to show similar peaks at 19.10°. For all PEG molecular weights, an identical peak was seen, but with varying intensities [24]. In PSf, the percentage of crystallinity decreases as crystallite size increases, and this trend holds true for the PEG of varying molecular weights. Research by Nasirian et al. [21] corroborates this conclusion by suggesting that an increase in PEG in mixed membranes could account for the PEG diffraction peak, which appears especially at 20°.

##### Scanning Electron Microscopy Analysis (SEM)

**Figure 5:**
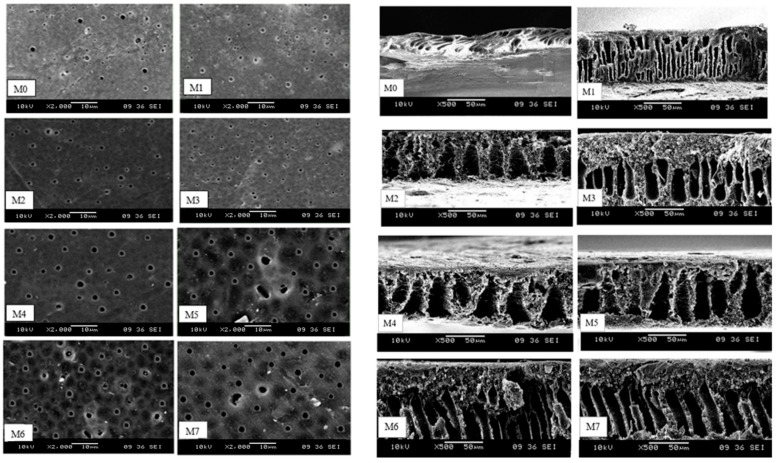
The SEM micrographs of PSF MMMs top surface at 2000× magnification and cross-section at 500× magnification. The obtained SEM images were represented the PSf MMMs morphology and structure from M0 till M7 in terms of the distribution of pores, surface density and formations of voids.

As can be seen in Figure 5, the PSf MMMs used in the dead-end filtration test underwent SEM analysis to characterize the top surface and cross-sectional morphology of each active site. PSf is hydrophobic in nature and fouls easily, making it the M0. The results demonstrated that the morphology and structure of the membrane, including the distribution of pores, were significantly affected by the addition of nanoparticles (NPs), specifically TiO_2_ and PEG, respectively. By altering the polymer membrane’s surface, we were able to test whether or not the addition of additives could increase the membrane’s hydrophilicity and decrease fouling tendency. The incorporation of non-solvent (DMAc) into the polymer dope solution was slowed. Macrovoids, sponges, and finger-like structures have all been linked to this phenomenon [25]. The top surface and cross-sectional morphology of all MMMs shown in Figure 5 are representative of all MMMs that were successfully fabricated. All of the NPs and polymer additives were spread out uniformly.

Figure 5 clearly shows cross-sectional images of each of the fabricated PSf MMMs; all of the fabricated membranes were highly porous with asymmetric configuration. Because of the addition of a constant number of NPs, the thickness of all fabricated membranes is constant at about 0.5 wt.%. As a result of the variable rate of exchange between solvent and non-solvent during the phase-inversion procedure, additives are incorporated into the membranes [26]. The membrane is primarily made up of interconnected finger-like structures with pores on the upper surface. The structure and morphology of the membrane may also indicate the presence of additives within the membrane, which were the primary source of pore formation, finger-like structure, and void formation of varying sizes. M0 had a similar finger-like structure to the pure PSf membrane but with a smaller pore size and the formation of an elongated void at the bottom layer. TiO_2_ and PEG’s hydrophilic nature increases mass transfer speed during solvent and non-solvent exchange, which contributes to the formation of larger pore channels. As a result of the significant void creation, the high-water flux matches the membrane shape [27]. To be clear, the addition of TiO_2_ and polymer additives lengthened the membranes on the bottom side, resulting in the formation of macrovoids as well.

However, the dispersion quality of TiO_2_ and PEG within the PSf matrix in MMMs is reflected in their cross-sectional morphology from M3 to M7. The SEM images demonstrate a more uniform cross-sectional morphology when PSf/PEG is added to TiO_2_. By combining the—OH of TiO_2_ with the—CH_2_ of PEG, we can ensure a strong interaction between the polymer and filler and prevent the formation of non-selective interface gaps. The sample polymer/particle interface quality further demonstrates that the polymer chains completely encircle the TiO_2_ particles [28]. Besides that, the M3 membrane had a shorter finger-like structure as well as large macrovoid dimensions and lengths because of the incorporation of NPs. The addition of PEG and TiO_2_ delayed the solvent exchange rate with coagulation bath, which resulted in a spongy structure formation at the underside layer of membrane. Therefore, the M6 and M7 membranes appeared to be more porous than other membranes [29]. Next, the M6 and M7 membranes had longer finger-like structures as well as a larger pore size compared to the M1 and M2 membranes due to the combination of both additives resulting in the increment of distribution of pores. The variation in the sublayer structures is intrinsically controlled by the demixing process, which is dependent on the phase-separation process’s combination of thermodynamic and kinetic factors. A recent paper highlighted the mechanisms of surface pores and macrovoid structures [18].

##### Water Contact Angle Analysis

**Figure 6:**
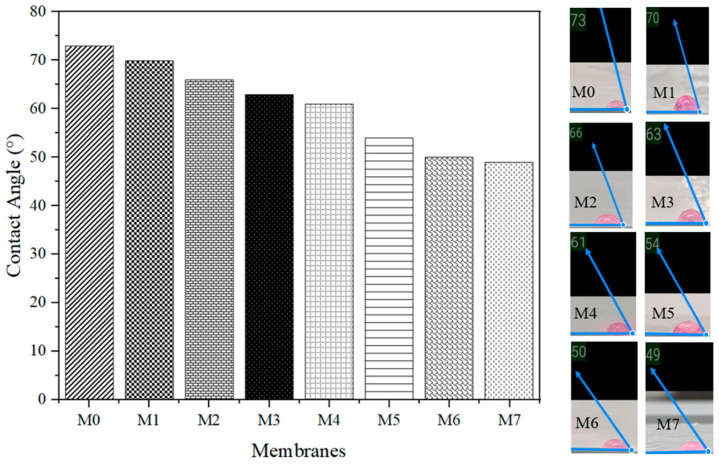
The contact angle of the fabricated PSf MMMs from M0 till M7. The graph depicted the contact angle measurement of membrane from highest angle of M0 membrane till the lowest angle of M7 membrane. The M0 with highest angle represented with lowest hydrophilicity whereas M7 with lowest angle represented with the highest hydrophilicity.

The membrane’s hydrophilicity and wettability can be inferred from its water contact angle [30]. Contact angle measurements were used to evaluate the hydrophilic impact of TiO_2_ and PEG on the matrix of PSf membranes. Additionally, Angle Meter Pro Plus software was used to calculate the water contact angle of membranes. The propensity of the membrane to foul is significantly affected by the contact angle of the water. If a hydrophilic membrane is able to generate a continuous hydration layer on its surface, this acts as a physical barrier, blocking foulants from adhering to the membrane [31]. Lowering contact angles indicate that surfaces are becoming more hydrophilic. Figure 6 displays images generated by Angle Meter Pro Plus software for the purpose of analyzing the contact angles of membranes after fabrication.

As seen in the trend from Figure 4, the contact angle decreased when the additive was added. The introduction of the hydrophilic additives such as TiO_2_ and PEG, which contains hydroxylic groups, to the membrane matrix [32] resulted in a gradual reduction in water contact angle values in the following sequence: M0 > M1 > M2 > M3 > M4 > M5 > M6 > M7. Contact angle measurement and hydrophilicity are inversely proportional to each other, such as when the contact angle increases, the hydrophilicity of MMMs decreases. The contact angle of the raw membrane was about 73° and dropped to 70° when TiO_2_ was added. Then, the same phenomenon occurred when PEG was added. This was proven from the study of Kamal et al. [32], with a pure PSf contact angle measurement of about 78°. In that case, it clearly shows that the measurement of the contact angle of PSf MMMs in this current study was in the range of previous studies [13]. Furthermore, the higher the molecular weight of PEG added, the contact angle was dropped from M3 to M7, which was 63° to 49°. This shows that the addition of PEG and TiO_2_ increases the wettability of the membrane as well as reduces the chance of fouling on the membrane [33].

##### Porosity Analysis

Membrane pore size indicates the median or mean size of the holes on a membrane surface. It also defines the particle size that the membranes can reject and describes the membrane’s performance [34]. Table 4 shows the overall porosity and mean pore size of M0, M1, M2, M3, M4, M5, M6, and M7 membranes. The pure PSf membrane, M1, exhibited the lowest porosity and mean pore size due to unfilled membranes with NPs and polymer additives compared with MMMs. This statement is confirmed by a previous study from Jyothi et al. [33], which states that the porosity of PSf was the lowest compared to other blended membranes, which was about 40%. This signifies that the porous value obtained for PSf MMMs in the current study is nearly in the range of the previous study.

M7 had the highest porosity among MMMs when compared to the others. This was due to the rapid demixing of the dope solution, which resulted in the rapid intrusion of filtered water into the membrane matrix [35]. This is because the interaction between the highest molecular weight PEG and TiO_2_ causes the membrane to form more pores. In other words, the addition of additives, particularly TiO_2_ and PEG, with low agglomeration tendency and formed even dope solution, resulted in a rapid phase-inversion process. The surface pore size increased in lockstep with the molecular weight of PEG. The M7 has the largest pore size of any cast membrane. M7’s larger surface pore size could be attributed to the highest molecular weight of PEG, which increased the hydrophilic nature of the membrane’s dope solution. According to the findings of Feng et al. [36], increasing the hydrophilicity of the cast solution accelerates the solvent/non-solvent exchange rate during phase inversion, resulting in an increase in the surface pore size of the membrane, as seen in M7.

Membrane porosity was calculated to quantify the morphological changes brought about by the addition of additives to the membrane matrix. Table 4 shows that as the number of NPs are added to MMMs, the overall porosity increases in the following order: M7 > M6 > M5 > M4 > M3 > M2 > M1. Evaporation rate, coagulation method, and solvent–polymer interaction were just a few of the many variables that influenced MMM porosity. During the phase-inversion process, macrovoids and channels were formed, contributing to the membranes’ increased porosity due to the fast movement of water molecules. The matrix of the membrane became more permeable and porous as a result.

The mean pore size of fabricated MMMs were well within the UF range of 1 to 50 nm [37]. The inclusion of NPs resulted in a slightly smaller mean pore size in M1 than in pure PSf membrane M0. This occurred because of the rapid demixing of PSf-NPs dope solution, as analyzed previously, that caused the development of bigger pores along with macrovoids. A slight increment in porosity was noticed in MMMs due to the presence of pore formers, which is the PEG. This observation was compatible with the cross-section of SEM images in Figure 5, which showed a broadened structure. Porosity was directly proportional to the hydrophilicity of membrane. Lastly, when porosity increases, the hydrophilicity of the membrane also increases. The overall effect is an increase in porosity as well as greater mean pore size of the MMMs, which is advantageous for the UF process.

### 3.2. Acetic Acid Removal Performance of Ultrafiltration PSf MMMs via Dead-End Filtration

#### 3.2.1. Pure Water Flux

**Figure 7:**
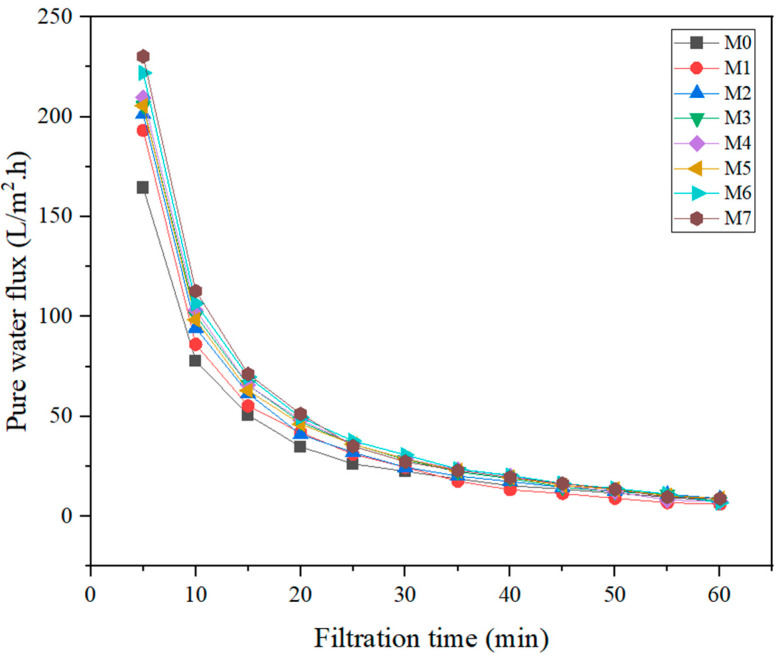
The PWF (J_WF1_) of different membranes against filtration time.

The dead-end filtration tests were performed on all the fabricated membranes at a 50 mg/mL concentration of acetic acid. From Figure 7, it was observed that the MMMs with NPs and polymer additives resulted in greater value in the PWF in conjunction with the pure PSf membrane. The PWF of pure PSf membrane, M0, had the lowest value of 130 L/m^2^.h in 50 mg/L of acetic acid as feed solute. The pure PSf membrane, M0, had the lowest permeability compared to those containing NPs due to the relatively high hydrophobicity character in the base PSf membranes and no macrovoids formation on the membrane surfaces.

The trend of PWF for fabricated PSf MMMs was declining from M0 to M7. M7 depicted the highest PWF compared to M0, which was the lowest. In accordance with the findings of Singh et al. [38], the PWF is 597.78 L/m^2^·h, and the maximum membrane flux ranged from 133.50 to 197.59 L/m^2^·h. The high permeability of MMMs corresponds with their remarkable hydrophilicity, as illustrated in Figure 7 and confirmed by the mean pore size in Table 4. As the contact angle decreased, the improving trend within the PWF was confirmed. It is directly proportional to the relationship between water permeability and membrane hydrophilicity. The permeation performance may also be promoted by the adsorption of water particles within the membrane matrix. The greatest amount of water flux of 170.1 L/m^2^·h for both concentrations resulted from the modified M7 membrane with PSf/TiO_2_/PEG 6000. M7 obtained the highest flux. At the same time, the water flux of the M6 membrane was recorded nearest to the value of M7, which is about 169.12 L/m^2^·h.

#### 3.2.2. Dilute Acetic Acid Flux (J_AA_) and Rejection (R_AA_)

The acetic acid rejection (RAA) of 50 mg/mL is shown in Table 5, which represents the RAA of the fabricated MMMs in filtering the AA solution. The modified membranes revealed a higher rejection rate compared to the pure PSf membrane. The AA rejection of the pure PSf membrane, M0, was 26.8%, which is the lowest compared to other membranes. Next, the AA rejection of the modified membrane from 41.4%, 53.4%, 58.8%, 68%, 85%, 90.2%, and 92.2% for M1, M2, M3, M4, M5, M6, and M7, respectively. The incorporation of NPs and polymer additives within the membrane matrix has impacted the rejection performance, which is expected due to the constant operating pressure.

Moreover, the size of the AA molecule was greater compared to water; hence, the water molecule was able to easily penetrate easily the membrane’s pore size. The phenomenon can be explained by the hydroxyl layer formation by high surface hydrophilicity during filtration. The hydrophilic layer repels AA molecules from penetrating the membrane, thus improving AA retained on the surface of the membrane. For the tested membrane of M7, the rejection capability was nearly 93 %; therefore, it may be confirmed that the exchange between selectivity and permeation has been increased, while the permeate flux was rarely influenced. The permeate flux was most slightly minimized after the primary cycle due to reversible fouling. Nevertheless, the permeate flux was retained consistently within the remaining cycles and demonstrated decent consistency as an assurance of the credibility of the M7 membrane. In this current analysis, M7 was justified as the well-performed membrane among others without compromising RAA, owing to its largest mean pore size as well as antifouling characteristics.

The trend of AA flux for fabricated PSf MMMs declined from M0 to M7. The slight drop in water flux was noticeable at 10 min due to the adsorptive separation AA, whereby the AA started to retain on the surface of the membrane. The J_AA_ for the pure PSf membrane, M0, was 126.71 L/m^2^·h. In doing so, incorporation of the NPs and polymer additive, J_AA,_ in 50 mg/mL rose considerably from 85.26 L/m^2^·h, 91.7 L/m^2^·h, 101.22 L/m^2^·h, 120.93 L/m^2^·h, 135.99 L/m^2^·h, 143.84, and 150 L/m^2^·h for M1, M2, M3, M4, M5, M6, and M7. At the end of the test, a relatively steady flux should be obtained for the reason that the equilibrium is attained within the deposition as well as sweeping [39].

### 3.3. Fouling Study

#### 3.3.1. Membrane Fouling Analysis

**Figure 8:**
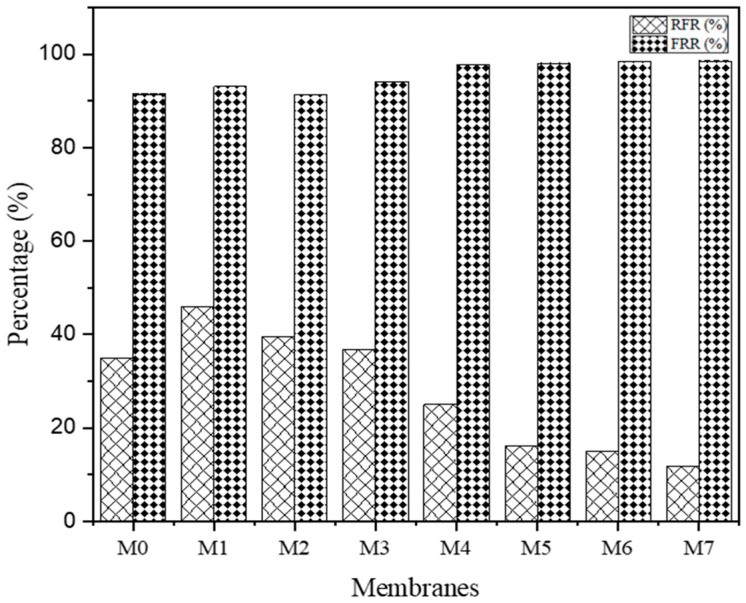
The antifouling properties of the fabricated membranes.

Among all of the fabricated membranes, the M7 of PSf/TiO_2_/PEG exhibited the highest flux recovery ratio (FRR) because of the presence of the hydrophilic layer support on the membrane matrix. The pure PSf membrane, M0, had the lowest FRR value in both concentrations, which revealed high exposure of membrane fouling. After dead-end cell filtration was complete, the membrane was exposed to 15 min of washing with running distilled water so as to get rid of the bonded foulant on the surface of the membrane. Then, MMMs were carried out as usual to measure the initial J_WF,_ followed by J_AA_ and J_WF2_. The relative flux reduction (RFR) was quantitatively calculated, and therefore, the hydraulic clean-up properties of the membrane may well be assessed by the flux recovery ratio (FRR), as shown in Figure 8. With the aim of achieving the best efficiency, the membranes illuminated great antifouling properties with low RFR and high FRR.

The ordinary method to examine membrane fouling mitigation is through FRR. This method may demonstrate irreversible fouling with the presence of adsorption of foulant on the surface of the membrane [40]. The higher value of FRR indicated a robust membrane resistance against fouling, and low value of RFR indicated a lower likelihood of membrane fouling. The lowest RFR fell into M5, M6, and M7, which indicated a lower chance of fouling. In order words, these three membranes had better antifouling ability compared to others. After membrane washing, the membrane permeability can be recovered by evaluating the cleaning efficiency by FRR value. M7 obtained the highest FRR value, about 98.5%, which implied high cleaning efficiency. Basically, a high number of studies have targeted the modification of membranes so as to achieve the feasible structure of antifouling properties of membranes. The pure PSf membrane, M0, seems to experience serious fouling activity because the hydrophobic properties of AA that created it are susceptible to AA fouling. Each of the MMMs exhibited mild fouling with higher reversible fouling. The M7 membrane displayed the highest percentage of FRR and the lowest percentage of RFR in the removal of AA, which confirmed the optimistic efficaciousness of the stated NPs and polymer additives within the improvement of fouling resistance characteristics of MMMs. This would reduce the cost of maintenance as well as sustainable filtration materials.

#### 3.3.2. Concentration Polarization (CP)

Identification of CP as a method demonstrated the adsorptive phenomenon in a membrane, which is rarely used in membrane research. Effort has been made to demonstrate that this study is subject to adsorptive separation. In general, concentration polarization (CP) refers to the concentration gradients that appear at a membrane–solution interface due to the preferential transport of specific species across the membrane. Previously, it has been thoroughly examined how this inherent permeability selectivity result relates to the mitigation of driving forces across the active layer of membranes for a number of membrane processes. In the ultrafiltration of macromolecules, CP varies depending on fouling. CP, as defined by IUPAC, is not associated with fouling and refers to the concentration profile with a higher level of solute closest to the upstream membrane surface (C_m_) compared to the more or less well-mixed bulk fluid distant from the membrane surface (C_b_) [41]. This is consistent with the argument made by researchers from another lab, who distinguished between concentration polarization, which occurs quickly and is reversible, and the fouling phenomenon, which occurs over time and is essentially irreversible. 

In this study, the AA solute retained on the membrane’s surface was greater than the AA feed concentration. The feed concentration was approximately 0.05 g/cm^3^, which is equivalent to 50 mg/mL. Table 6 depicts the concentration polarization that occurs on the surface of the membrane, resulting in solute retention on the surface of MMMs. The reason for the accumulation of AA solute on the membrane surface is that the molecular weight cut-off (MWCO) of AA was greater than the membrane matrix. In that case, the water molecule passed through the membrane surface more easily than the AA solute, which is why M7 has a higher PWF and J_AA_ than M0. M7 had the most pores, while M0 had the fewest. As a result, water can easily permeate the MMMs. It is worth noting that this phenomenon is known as adsorptive MMMs because the AA is retained on the membrane’s surface.

**Figure 9:**
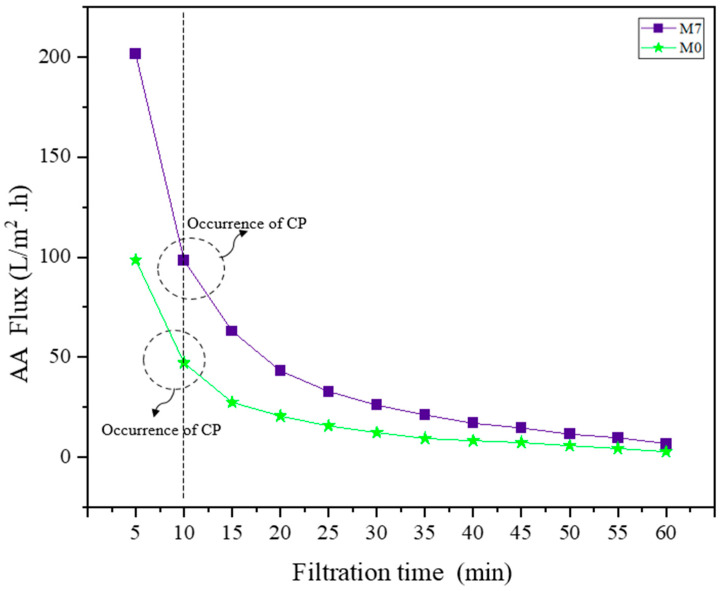
Sudden flux decline in the M0 and M7 membranes at 10 min. The dotted line of the M0 and M7 membranes showed the AA flux declining at every interval in the 60 minutes timeframe.

Figure 9 depicts the relationship between the flux decline in M0 and M7 in an hour of dead-end filtration test. Both M0 and M7 depicted the occurrence of CP after 10 min of the permeation test. This clearly shows that the AA was separated on the surface of the membrane. For M0, there was a slightly obvious flux decline compared to M7, which showed a drastic decline in flux after 10 min. This is because the pores distributed on the surface of the M0 membrane were in fewer amounts in comparison to M7. The presence of PEG and TiO_2_ helps with the formation of pores as well as the hydrophilic layer formed on the M7 membrane [42]. Therefore, there is a lower chance for the deposition of foulants on the top of the membrane instead of the AA molecules from the feed solution retained membrane. To summarize, there are significant distinctions between CP and fouling. CP involves molecules that are still in solution, whereas fouling by macromolecules involves molecules that are no longer in solution.

## 4. Discussion

**Figure 10:**
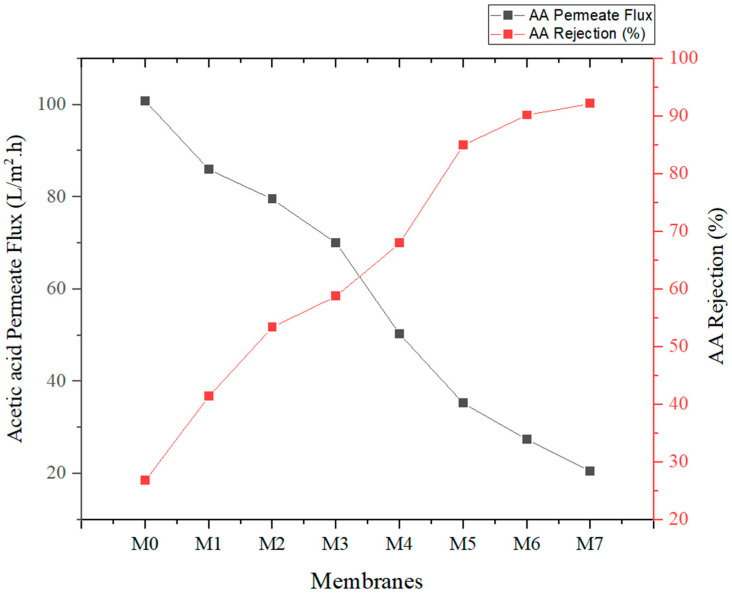
Relationship between pure water flux and AA permeate flux.

The association between AA permeate flux (J_AA_) and AA rejection (%) from M0 to M7 is depicted in Figure 10. AA permeate flux is inversely proportional to the AA rejection (%); this depicts that the combination of PSf MMMs with TiO_2_ and PEG leads to the highest rejection in the M7 membrane in comparison to the permeation of AA. PEG, which can be used as a non-solvent pore-generating element in casting solutions containing PSf membrane and solvents such as dimethylacetamide (DMAc), is the principal cause of the increase in PWF and J_AA_ [43]. As a second material, PEG modifies the PSf ultrafiltration membrane’s structure and performance. Polymer membrane performance is significantly affected by the amount of PEG employed and its molecular weight.

The number and area of pores in membranes increase as the molecular weight of PEG increases. In addition to its high porosity, a membrane with a higher molecular weight PEG possesses a better pure water flux (PWF) compared to AA permeate flux due to the solute molecular weight cut-off and hydraulic permeability. The use of a PEG component can increase membrane hydrophilicity [44]. When developing membranes with improved mechanical characteristics, the quantity of PEG and its molecular weight should be taken into account [45]. This is due to the fact that the mechanical characteristics were influenced by the wrong dosage and PEG molecular weight. This is proof that the presence of PEG increases water permeability due to the availability of mean pore size from 0.025 μm to 0.5 μm, which enables water molecules to pass through the membrane while acetic acid is trapped on the membrane’s surface. To summarize, the introduction of PEG 400 in M2 resulted in a significant increase in PWF from M1 to M2, resulting in a larger dispersion of pores. Following that, the fluxes continuously increased from M2 to M7. The inclusion of NPs and polymer additives improved the porosity, mean pore size, and water uptake. NPs play an essential role in broadening the finger-like pore size as well as the interconnectivity across the membrane thickness, which reduces membrane hydraulic resistance and enhances membrane flux [46]. The factor for the enhancement of mean pore size is because of the presence of PEG with higher molecular weight. Chew et al. [46] states that the higher the molecular of PEG, the higher the number of pores formed on the surface of the membrane. Furthermore, the water flux increased gradually from M3 to M7 with an increase in the molecular weight of PEG. 

## 5. Conclusions

To sum up, the three main goals of dilute acetic acid (AA) separation using fabricated polysulfone mixed matrix membranes (PSf MMMs) were achieved and validated in comparison to the commercial chemical method. First and foremost, a phase-inversion process was used to successfully fabricate PSf MMMs by incorporating titanium dioxide (TiO_2_) and polyethylene glycol (PEG). The results of analyzing how this additive affected the PSf MMMs’ porous structure, morphology, and surface properties were presented. PSf/TiO_2_/PEG membranes successfully matched the performance and antifouling properties of pure PSf, and the concentration polarization of the membranes was also compared. Chemical and physical analysis yielded the expected outcomes. Across the board, the absorption range of functional groups for PSf, TiO_2_, and PEG was discovered, indicating that the resulting FTIR spectrum was positive. The sample’s intense X-ray diffractometer (XRD) peaks show that the polymer and nanoparticles formed were crystalline, and the sample’s broad diffraction peaks show that the crystallites formed were extremely small in size. The XRD pattern shows that as the concentration of TiO_2_ increases in PSf, the peak intensity and full width at half maximum (FWHM) decrease. Morphological analysis of the synthesized PSf MMMs revealed the pore distribution and membrane roughness. When compared to other PSf MMMs, M7’s membranes displayed the highest pore count. Hydrophilicity is correlated with membrane pore size, so an increase in pore distribution correlates with an increase in hydrophilicity. Since the presence of PEG 6000 results in an increase in membrane pores, the M7 membrane has the highest hydrophilicity. Inorganic fillers with a wide range of characterization were used to improve PSf membranes, yielding the desired results. The fluxes of both pure water (PWF) and acetic acid (J_AA_) showed a consistent pattern of growth from M0 to M7. The results demonstrated that the NPs and polymer additive improved the diffusivity and overall permeability of the small molecules without compromising selectivity in M7. As solubility and diffusivity improved, permeability rose as a by-product. M7’s effectiveness as an antifouling agent is demonstrated by a significant reduction in both the reversible and irreversible fouling of AA. By incorporating NPs with hydrophilic functional groups such as hydroxyl and carboxylic acid into this study’s phase-inversion procedure, researchers were able to improve the interaction between the water flow and the NPs. According to the findings, the PSf/TiO_2_/PEG membrane is the best antifouling membrane, which could lead to exciting new applications for membrane technology. 

## Figures and Tables

**Table 1:** Dope solution formulations.

Label of the Membrane	UF Membrane	PSf wt.%	DMAc wt.%	Mixed Matrix Membrane (MMM)	
				TiO_2_ (wt.%)	PEG (wt.%)
M0	PSf membrane	16	84	-	-
M1	PSf + TiO_2_ membrane	16	83.5	0.5	-
M2	PSf + PEG 400 membrane	16	79	-	5
M3	PSf + TiO_2_ + PEG 600 membrane	16	78.5	0.5	5
M4	PSf + TiO_2_ + PEG 1000 membrane	16	78.5	0.5	5
M5	PSf + TiO_2_ + PEG 1500 membrane	16	78.5	0.5	5
M6	PSf + TiO_2_ + PEG 4000 membrane	16	78.5	0.5	5
M7	PSf + TiO_2_ + PEG 6000 membrane	16	78.5	0.5	5

**Table 2:** Reaction from the incorporation of PSf/PEG and PSf/PEG/TiO_2_ membrane.

Possible Interaction between Polymer and Additives	Description
** 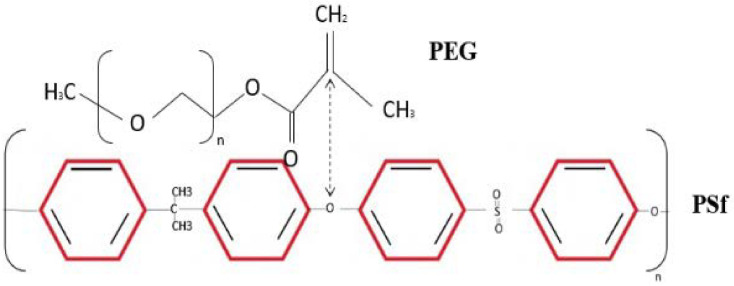 **	Reaction of PSf and PEG.
** 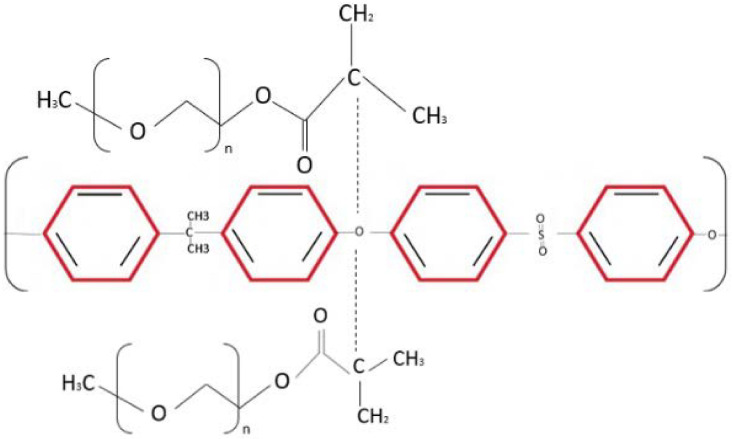 **	The carbon oxygen reaction between PSf and PEG molecules blended in membrane.
** 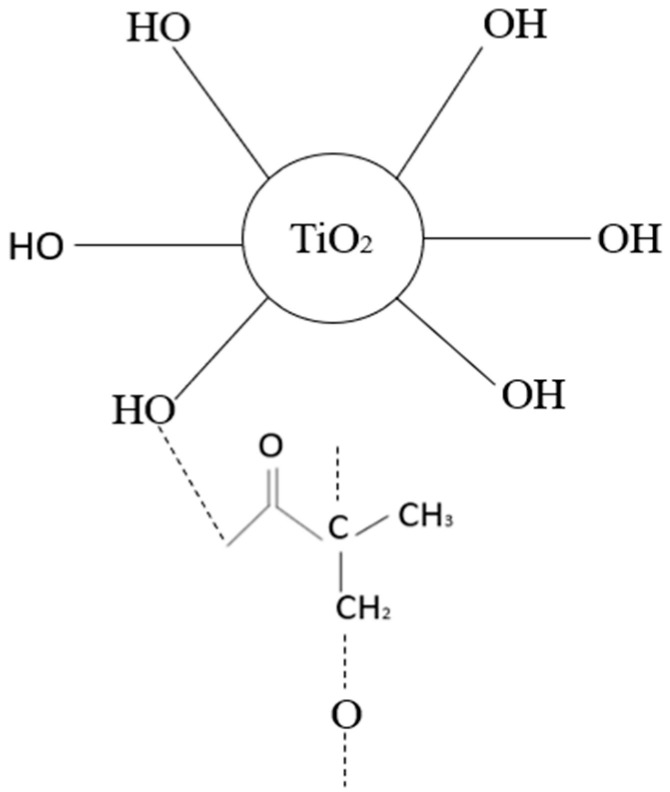 **	Carbon oxygen stretching between polymer chain and TiO_2_ particles in membrane matrix.

**Table 3:** Calculated interplanar spacing and crystallite size of polymer and additives.

Peaks	Peak Position, 2θ	Intensity	Interplanar Spacing d (Å)	Crystallite Size (nm)
Peak 1	22.45	11,961	1.499	27.176
Peak 2	34.109	1936	2.25	25.237
Peak 3	47.57	12,547	3.104	22.196
Peak 4	19.10	2759	1.277	30.05
Peak 5	19.10	2831	1.277	50.543
Peak 6	19.10	3407	1.277	48.659

**Table 4:** The overall membrane porosity and mean pore size of fabricated of PSf MMMs.

Membrane	Porosity (%)	Mean Pore Size (nm)
M0	32.9	25.02 ± 0.2
M1	38.5	29.02 ± 0.2
M2	46.1	31.45 ± 0.3
M3	62.9	32.39 ± 0.4
M4	68.2	33.57 ± 0.4
M5	68.3	34.65 ± 0.2
M6	68.8	44.92± 0.2
M7	70.7	50.63 ± 0.4

**Table 5:** The initial PWF, AA retentate and final PWF, percentage rejection of AA, and concentration of rejected AA from the membrane.

Membranes	Initial PWF (J_wF1_) (L/m^2^·h)	Diluted AA Permeate (J_AA_) (L/m^2^·h)	Diluted AA Retentate (R_AA_) (L/m^2^·h)	Final PWF (J_wF2_) (L/m^2^·h)	The Concentration of Permeated Diluted Acetic Acid (Retentate) (mg/mL)	Retentate (%) of Diluted AA on Membrane
M0	130	100.82	70.41	126.71	36.6	26.8
M1	131.2	85.96	85.26	130.1	29.3	41.4
M2	151.52	79.52	91.7	144.18	23.3	53.4
M3	160.22	70	101.22	150.75	20.6	58.8
M4	161.14	50.27	120.93	154.11	16	68
M5	162	35.27	135.99	156.16	7.5	85
M6	169.12	27.40	143.84	160.96	4.9	90.2
M7	170.1	20.50	150	164.73	3.9	92.2

**Table 6:** The concentration of AA on the surface of the membrane.

Types of Membrane	Concentration Polarization of AA, C_m_(g/cm^3^)	Concentration of Permeated AA, C_p_ (g/cm^3^)
M0	0.065	0.036
M1	0.072	0.029
M2	0.079	0.023
M3	0.082	0.02
M4	0.087	0.016
M5	0.097	0.0075
M6	0.100	0.0049
M7	0.101	0.0039

## Data Availability

The data presented in the study are openly available in the Results and Discussion sections of this study.
